# Effect of environmental factors on gene alterations in complex diseases: simulation study using Gene-Environment iNteraction Simulator 2

**DOI:** 10.1186/1471-2164-15-S2-P47

**Published:** 2014-04-02

**Authors:** S Abdalla, Y Al-Hadeethi, E K El-Shewy

**Affiliations:** 1 Department of Physics, Medical-Physics Division, Faculty of Science, King Abdulaziz University, Jeddah, KSA; 2 Theoretical Physics Research Group, Physics Department, Faculty of Science, Mansoura University, Mansoura 35516, Egypt

## Background

The gene alterations and etiological pathway of complex diseases such as cancer, Down-syndrome, obesity, etc is highly affected with the interactions between genes and environmental factors. To handle a wide variety of complex situations (such as genes and environmental factors) including certain interactions between variables is poorly investigated, in particular if continuous variables will be involved. Simulations with realistic data sets with gene-environment interactions and gene- gene interactions which affect the risk of a complex disease are a convenient and useful way to characterize the validity of statistical-models. In a previous work [1], we have examined the interaction between one gene and one environmental factor as a function of time. In the present study, the gene-environment iNteraction simulator (GENS2) has been used to simulate interactions through two genetic and one environmental factor which permit, also, for study the same effect on epistatic interactions.

## Materials and methods

We have simulated interactions among two genetic and one environmental factor using the Gene-Environment iNteraction Simulator 2 (GENS2). It is released under the general public license version3.0 [2].This software generates data which allows for epistatic interactions; it imposes no limitations either on how many simulated-individuals or on number of factors (environmental/genetic) which should be taken into consideration. Also, we present a mathematical- model to examine the case of two possibly interaction disease predisposing loci. Only one environmental factor has been chosen to interact with two disease predisposing loci with the assumption that: 1- the genetic loci are assumed to have insensitive effects, i.e. no epistasis and they can easily interact following an epistatic way. 2- The disease risk is highly affected with the genetic alterations (either directly- in linear manner or by modifying the effect of the environment). 3- Also, the disease predisposing is not in linkage disorder.

## Results

**Figure 1:**
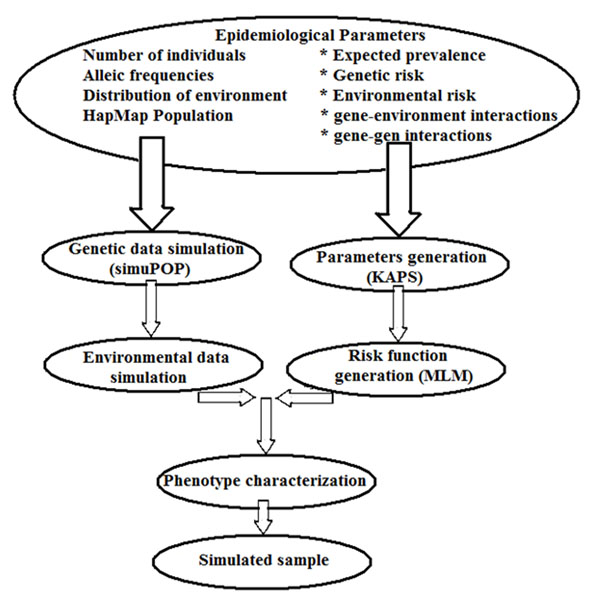
Chart of the steps that have been used to simulate a complex disease in a population using the simuPOP and GENES2 systems.

Realistic data of linkage disequilibrium have been used as input source with Gene-Environment-iNteraction-Simulator2 (GENS2) to simulate gene-environment interactions and gene- gene interactions with no restriction either on the number of individual or number of non-predisposing. The efficiency of GENS2 simulator can be increased when the input parameters are stated as standard epidemiological values. GENS2 uses advantage of modules and operators given by the simuPOP simulation-environment [2]. This simulator-software can be used with a command line interface as shown in Fig. 1. On a population of about one thousand cases and one thousand controls with two disease predisposing loci a single marker analysis has been used as first examination with no epistasis and an additive gene-environment interactions model. Using a model where the disease risk should equal to the genetic factor, each marker has been tested (corresponding to the status) with a logistic-regression analyses. It is worth noting that non causative markers in linkage disease with the two disease predisposing loci show an important association which nearly depends on disease predisposing loci as r2.

## Conclusions

Gene- environment interactions and gene- gene interactions could be adequately identified and represented using GENS2-simulator giving rise to statistical data.

## References

[B1] Abdalla S Al-Hadeethi Y Gene's alternations with exposure time of environmental factors Gene 2013 15 2 256 60 10.1016/j.gene.2013.06.065. Epub 2013 Jul 13 23860326 10.1016/j.gene.2013.06.065

[B2] Amato R Pinelli M D'Andrea D Miele G Nicodemi M Raiconi G Cocozza S Novel approach to simulate gene–environmental interactions in complex diseases BMC Bioinforma 2010 15 8 1 9 10.1186/1471-2105-11-8 PMC2824681 20051127

